# Geological, land use and biological influences on carbon cycling and CO_2_ degassing in the Danube River

**DOI:** 10.1038/s41598-026-59715-0

**Published:** 2026-06-26

**Authors:** Jan Maier, Johannes A. C. Barth

**Affiliations:** Department of Geography and Geosciences, Geozentrum Nordbayern, Friedrich-Alexander-Universität Erlangen-Nürnberg, Schlossgarten 5, 91054 Erlangen, Germany

**Keywords:** Dissolved inorganic carbon (DIC), *δ*^13^C_DIC_ stable isotope analyses, *p*CO_2(aq)_, Groundwater-surface interactions, Freshwater research, Weathering, CO_2_ fluxes, C_3_/C_4_ isotope mass balance, Biogeochemistry, Climate sciences, Ecology, Ecology, Environmental sciences, Hydrology

## Abstract

River systems form critical interfaces in the terrestrial-atmospheric carbon cycle, yet basin-scale constraints on the spatial and seasonal controls of inorganic carbon dynamics and CO_2_ exchange remain limited. We present a multi-seasonal longitudinal assessment of dissolved inorganic carbon (DIC), its stable isotopes (*δ*^13^C_DIC_), and aqueous CO_2_ partial pressure (*p*CO_2(aq)_) along the entire Danube River. Five sampling campaigns captured seasonal variability along the main stem and major tributaries of this geologically diverse basin. DIC comprised > 90% of total carbon, showing limited longitudinal and seasonal variability (0.3 to 5.3 mmol L^− 1^), with highest concentrations in the upper Danube due to groundwater inputs and carbonate weathering, followed by downstream homogenization. In contrast, *δ*^13^C_DIC_ increased downstream from − 13.3 to -8.7‰, indicating cumulative CO_2_ degassing, with deviations linked to tributary inflows and biological activity. C_4_ plant inputs may have contributed up to 27.2% to the *δ*^13^C_DIC_. River *p*CO_2(aq)_ ranged from ∼260 to 3,770 µatm, predominantly supersaturated, with CO_2_ fluxes of 0.975–1.993 Gg C d⁻¹, confirming the Danube as a persistent CO_2_ source. Near-equilibrium conditions in spring and summer coincided with enriched *δ*^13^C_DIC_, consistent with episodic photosynthetic uptake. These results reveal how groundwater inputs, in-stream photosynthesis, and air-water exchange regulate inorganic carbon dynamics and CO_2_ emissions in large rivers.

**Supplementary Information:**

The online version contains supplementary material available at 10.1038/s41598-026-59715-0.

## Introduction

Inland waters act as key regulators of the global carbon cycle by regulating exchanges between terrestrial ecosystems, the atmosphere, and the ocean^1, 2^. Far from being passive transport pathways, rivers actively transform, store, and emit carbon through coupled hydrological, geochemical, and biological processes and are known to return substantial amounts of terrestrially mobilized carbon to the atmosphere as CO_2_^3, 4, 5, 6^. This concept of rivers as “mobile reactors” underscores the importance of fluvial networks in the global carbon budget^7, 8, 9^.

Active carbon transformation in large river systems is complex because they connect diverse geological units, climate regimes, and land use domains along extended flow paths. Basin-scale studies of various large river systems including the Amazon, the Mississippi, and the Mekong River, revealed persistent supersaturation of CO_2_ relative to atmospheric equilibrium^10, 11, 12, 13^. However, associated degassing may become interrupted under conditions of enhanced primary production or altered hydrological conditions, shown for instance in the Mississippi and St. Lawrence Rivers^14, 15^. Despite these advances, integrated assessments that explicitly resolve both the spatial and the seasonal variability in river carbon dynamics across entire river systems remain so far hardly investigated. This is particularly true for the Danube River, where apart from earlier studies limited to the upper basin^16, 17^, no comprehensive basin-wide assessments exist, even though the Danube represents one of Europe’s most important waterways.

To address this knowledge gap, carbon tracers that integrate catchment-scale inputs with in-channel processing and air-water exchange along the entire river continuum offer integrative tools. For instance, dissolved inorganic carbon (DIC) dominates the carbon pool in most river systems and therefore is a good integrative tracer for freshwater carbon cycling^18^. In the Danube River, DIC accounts for more than 90% of the total dissolved and particulate carbon load^19^. and also buffers pH, sustains aquatic primary production, supports biogenic calcification, and closely couples to other elemental cycles, such as that of oxygen^20^. All these functions are largely mediated by bicarbonate (HCO_3_^−^), which dominates the DIC pool under the circumneutral pH conditions (∼6–8.5) that typical of most natural waters^21^ including the Danube. Combination with its stable isotope ratios (*δ*^13^C_DIC_, expressed in ‰) enables the identification of dominant carbon sources and transformation pathways, including chemical weathering, and biological uptake through photosynthesis. Each of these processes causes characteristic isotopic changes, that can be disentangled via *δ*^13^C_DIC_ constraints^22, 23, 24^. How DIC relates to CO_2_ degassing and other land use factors can also be constrained by *δ*^13^C_DIC_ investigations.

While DIC concentrations and *δ*^13^C_DIC_ isotope values integrate information on carbon sources and in-stream transformation, they do not directly quantify CO_2_ degassing to the atmosphere. This process is best constrained by the partial pressure of carbon dioxide in the water column (*p*CO_2(aq)_) and associated fluxes (*F*_CO2_). Here *p*CO_2_ indicates which parts of the river act as net sources or sinks for atmospheric CO_2_ in seasonal variations^25, 26^. By concept, riverine *p*CO_2(aq)_ arises from the balance of groundwater and upstream terrestrial inputs, in-stream photosynthesis, and degassing. It therefore directly links hydrological inputs with aquatic carbon cycling and atmospheric CO_2_ emissions^27, 28, 29^. Because *p*CO_2_ is governed by carbonate equilibrium and temperature-dependent gas solubility, its variability can differ from changes in bulk DIC concentrations and its associated *δ*^13^C_DIC_ values. This individual behavior renders *p*CO_2_ and associated fluxes particularly sensitive to seasonal shifts in discharge, temperature, and biological activity.

Resolving the coupled controls on riverine inorganic carbon dynamics and CO_2_ exchange can reflect continental trends when observed along large river systems, were upstream inputs and in-channel processes cumulate along the flow path. The Danube River presents a good case study for such investigations because it drains a geologically diverse catchment spanning alpine, karstic, and lowland regions and further receives major tributaries such as the Inn, Sava and Tisa. With this setting it integrates multiple hydrogeological and ecological processes along its course. This diversity provides a framework for evaluating how groundwater inputs with alternating land use, photosynthesis, and degassing collectively shape riverine carbon dynamics.

In this study, we present a multi-seasonal and basin-scale assessment of DIC concentrations, *δ*^13^C_DIC_, and *p*CO_2(aq)_. It showcases a new dataset with 327 measurements along the Danube River continuum over more than one hydrological year. By combining longitudinal sampling with seasonal observations, our study aims to (i) identify the dominant lithological and hydrogeological controls governing riverine DIC sources and sinks, (ii) disentangle influences by groundwater inputs, biological uptake, and CO_2_ degassing to DIC and *δ*^13^C_DIC_ patterns, and (iii) assess how weathering, photosynthesis and CO_2_ degassing regulate *p*CO_2(aq)_ dynamics in a large temperate river system. By integrating chemical, stable isotope, and hydrological tracers, this study provides new insights into carbon cycling in the Danube River and offers a framework applicable to other large continental rivers.

## Methods

### Study area

The Danube River is a hydrologically and geologically diverse river system in Europe. It extends over 2857 km with a mean annual discharge of 6486 m^3^ s^− 1^, the river drains a catchment area of 807 827 km^2^ and crosses ten countries in central and eastern Europe^30^. The basin encompasses a wide range of climatic, lithological and geomorphic settings, including silicate-dominated headwaters, carbonate-rich karst terrains, alpine sub-catchments, and extensive lowland floodplains. This diversity of landscapes in the basin offers a natural laboratory for investigation of the river carbon cycle at the basin-scale.

The river originates in the Black Forest, where the Brigach and Breg headwater streams drain predominantly silicate lithologies and merge to form the Danube. Downstream of this confluence, the river receives input from major alpine and lowland tributaries including the Lech, Isar, Inn, Enns, March, Váh, Dráva, Tisa, Sava, and Siret. These tributaries drain various geological domains with both silicate and carbonate lithologies and exhibit markedly different hydrological regimes with variations in relief, climate, and geology across the basin^30, 31, 32, 33^.

The Danube basin hosts approximately 83 million inhabitants and represents one of Europe´s most important freshwater resources with water for domestic and industrial purposes, agriculture, and ecological needs^34, 35^. Owing to its transboundary nature and environmental significance, the river has been the focus of extensive international monitoring and management initiatives, including the Joint Danube Surveys coordinated by the International Commission for the Protection of the Danube River (ICPDR).

**Fig. 1:**
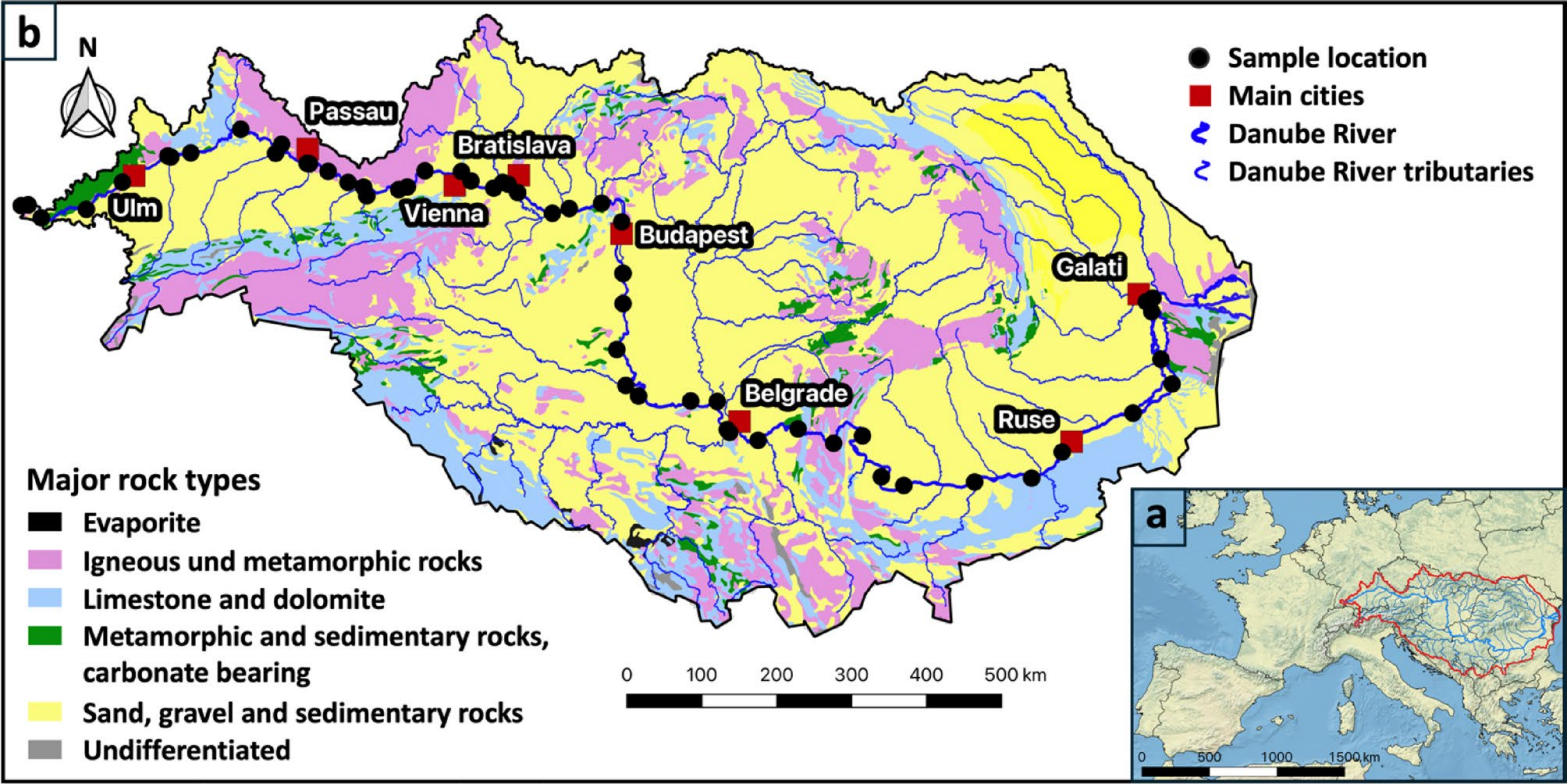
Study area and geological setting of the Danube River catchment. (**a**) Overview map of Europe with an outline of the Danube catchment (red). (**b**) Geologic overview with major rock types. Outline data for the catchment were provided by the ICPDR (data source ICPDR, 2025, last access: 16 October 2025). The following tributaries were sampled at their entry into the Danube: Brigach, Breg, Lech, Isar, Inn, Enns, March, Váh, Dráva, Tisa, Sava, and Siret. The map was created via QGIS v 3.28.3 with raster data from Natural Earth Data (version 3.2.0, https://www.naturalearthdata.com/downloads/10m-raster-data/10m-cross-blend-hypso/, last access: 16 December 2025), shapefiles from geoBoundaries (https://www.geoboundaries.org/globalDownloads.html, last access: 16 October 2025), and lithology from geodatenkatalog (https://gdk.gdi-de.org/geonetwork/srv/api/records/8329bc7e-d71a-4927-9240-9079f8608548, last access 16 October 2025).

To capture the spatial and seasonal variability inherent to this heterogeneous system, we conducted five sampling campaigns between 2023 and 2024 along the entire Danube main stem and selected inflows of major tributaries (Brigach, Breg, Lech, Isar, Inn, Enns, March, Váh, Dráva, Tisa, Sava and Siret). The campaigns covered summer (July 2023), fall (late October – early November 2023), winter (February 2024), spring (April 2024), and late summer (late August – early September 2024) periods. Each campaign covered between 54 and 89 sites along the Danube (Fig. 1). Site coordinates were determined in the field via Google Maps and were verified with a Garmin eTrex HC-series GPS unit. Discharge data for the Danube and major tributaries were provided by the ICPDR (https://www.danubehis.org, last access: 11 March 2025; ^36^.

### Field methods

Field methods are described in detail in Maier et al. ^20^. Briefly, water samples were collected with a narrow-mouth 2-L polyethylene sampling bottle that was attached to a weight and submerged 1 m below the water surface to obtain representative, well-mixed water from the river. The selected sampling depth helps minimize the influence of evaporation or precipitation. For determination of *p*CO_2_ we rely on bulk water chemistry, which notably ignores gradients of *p*CO_2_ at the air-water interface. However, we assume the river to be well mixed due to turbulent flow.

For DIC analyses and their corresponding *δ*^13^C values, water samples were filtered through 0.45 μm pore size disposable polyethersulfone (PES) syringe filters into 40-mL amber vials. These vials were pre-poisoned with 20 µL of a saturated HgCl_2_ solution to avoid any secondary biological activity after sampling. After gas free filling they were sealed with a rubber/PTFE septum and stored at 4 °C in the dark until further analyses.

Water temperature (*T*) and pH were measured in situ via a multiparameter instrument (HQ40d, HACH™, Loveland, CO, USA), calibrated daily. The analytical precision (± 1σ) was ± 0.1 units for T and pH. Unfiltered water samples were titrated in situ with a 1.6 N H_2_SO_4_ to quantify the total alkalinity (TA) that corresponded to the HCO_3_^−^ concentration. Replicate titrations indicated a measurement uncertainty of 2% (± 1σ).

### Laboratory methods, stable isotope measurements

The concentrations and stable isotope ratios of DIC were determined by a TIC TOC analyzer (Aurora 1030 W, OI Analytical, College Station, Texas, USA) that applied a wet oxidation method with phosphoric acid and persulfate in two successive steps. The instrument was operated in continuous flow mode and coupled to an isotope ratio mass spectrometer (IRMS, Delta V plus, Thermo Scientific). Further analytical details are provided in St-Jean^37^ and van Geldern et al. ^38^.

All DIC isotope ratios are expressed relative to an international standard according to the *δ* notation:

1 $$\:{\delta\:}^{13}\mathrm{C}=\frac{{\frac{13}{12}}_{{R}_{s}}}{{\frac{13}{12}}_{{R}_{r}}}-1$$

where $\:{\frac{13}{12}}_{{R}_{s}}$ is the carbon isotope ratio of the heavy to light isotopes in the sample and where $\:{\frac{13}{12}}_{{R}_{r}}$is the ratio of the reference standard (Vienna Pee Dee Belemnite, VPDB, ^39^. All the isotope values were multiplied by 1000 to express them in per mil (‰). The isotope data were corrected for instrumental drift and linearity according to van Geldern et al. ^38^. Standard deviation (1σ) was better than 5% for DIC concentrations and ± 0.2‰ for DIC isotopes based on controls and replicate measurements.

### Calculations

The partial pressure of carbon dioxide in the water column (*p*CO_2(aq)_) was calculated according to:

2 $$\:p{CO}_{2}\:=\frac{{HCO}_{3}^{-}\:\times\:\:{H}^{+}}{{K}_{H}\:\times\:\:{K}_{1}}$$

where [H^+^] = 10^− pH^, *K*_1_ is the temperature-dependent first dissociation constant of carbonic acid (H_2_CO_3_), and *K*_*H*_ is Henry´s law constant in mol L^− 1^ atm^− 1^^40, 41^.

All *p*CO_2(aq)_ values were computed via measured field parameters and equilibrium constants corrected for in situ temperature. Results were further cross-checked with calculations performed via the code PHREEQC^42^. The mean relative deviation between the direct calculations and PHREEQ-C-derived *p*CO_2(aq)_ values was ≤ 5% and confirmed the robustness of the applied approach. Mean values of both methods were calculated, with standard errors smaller than the symbol size shown in Fig. 4. Note that our technique ignores spatial and temporal disequilibria across water surface because PHREEQ-C only provide values for thermodynamic equilibria. Therefore, our calculations must be seen as best approximations.

Endmember values for *δ*^13^C of soil CO_2_ were derived from bulk organic matter input signals of -28.8‰ and  -12.5‰ for C_3_ and C_4_ plants^43^. Following Cerling et al. ^44^, both values were assumed to have become enriched by + 4.4‰ due to diffusion. According to Myrtinnen et al. ^45^ and Mayer et al. ^46^ a further isotope enrichment of + 8.5‰ occurs due to equilibration between the soil CO_2(g)_ the DIC species (CO_2(aq)_*, HCO_3_^−^ and CO_3_^2−^) at average groundwater temperatures of 12 °C and a pH of 7.5 and yields endmember *δ*^13^C_DIC_ input signal of -15.6‰ for C_3_ plants and + 0.4‰ for C_4_ plants. These endmembers represent idealized equilibrium conditions and therefore provide a first-order approximation of groundwater input signals.

To quantify the relative contributions of these sources, a two-endmember isotope mass balance was applied:

3 $$\:{{\delta\:}^{13}\mathrm{C}}_{\mathrm{m}}=\frac{{{\delta\:}^{13}\mathrm{C}}_{\mathrm{C}3}*X+{{\delta\:}^{13}\mathrm{C}}_{\mathrm{C}4}*Y}{X+Y}$$

where *δ*¹³Cₘ represents the apparent input *δ*¹³C_DIC_ value derived from Miller-Tans plots for each season (-11.2, -13.7, -13.3, -14.7, and  -15.4‰ for summer, fall, winter, spring, and late summer, respectively; Fig. 5). *δ*¹³C_C3_ and *δ*¹³C_C4_ correspond to the defined endmembers (-15.6‰ and + 0.4‰), where X and Y denote their relative contributions.

Assuming X + Y = 100% and substituting Y = 100 - X, Eq. (3) can be rearranged to:

4 $$\:\mathrm{X}=\frac{{{(\delta\:}^{13}\mathrm{C}}_{\mathrm{m}}*100\:-\:{{\delta\:}^{13}\mathrm{C}}_{\mathrm{C}4}*100)}{{{(\delta\:}^{13}\mathrm{C}}_{\mathrm{C}3}-\:{{\delta\:}^{13}\mathrm{C}}_{\mathrm{C}4})}$$

The resulting values provide an estimate of the relative contribution of C_3_-derived carbon to riverine DIC, while the C_4_ fraction is obtained as the residual (100 - X).

We note that the *δ*¹³Cₘ values derived from Miller-Tans analysis represent apparent source values that may integrate both groundwater inputs and in-stream processes such as CO₂ evasion. Furthermore, the C_4_ endmember reflects a theoretical upper bound assuming complete equilibration, and thus calculated C_4_ contributions should be interpreted as maximum estimates.

### Statistical analyses

All statistical analyses were conducted in R (v.4.3.2; ^47^) using the lm() function to create the linear model and anova() for variance analysis. The coefficient of determination (R²) was reported to evaluate the proportion of variance in Miller-Tans plot (^48^ ;Figure 5a, b).

## Results

**Fig. 2:**
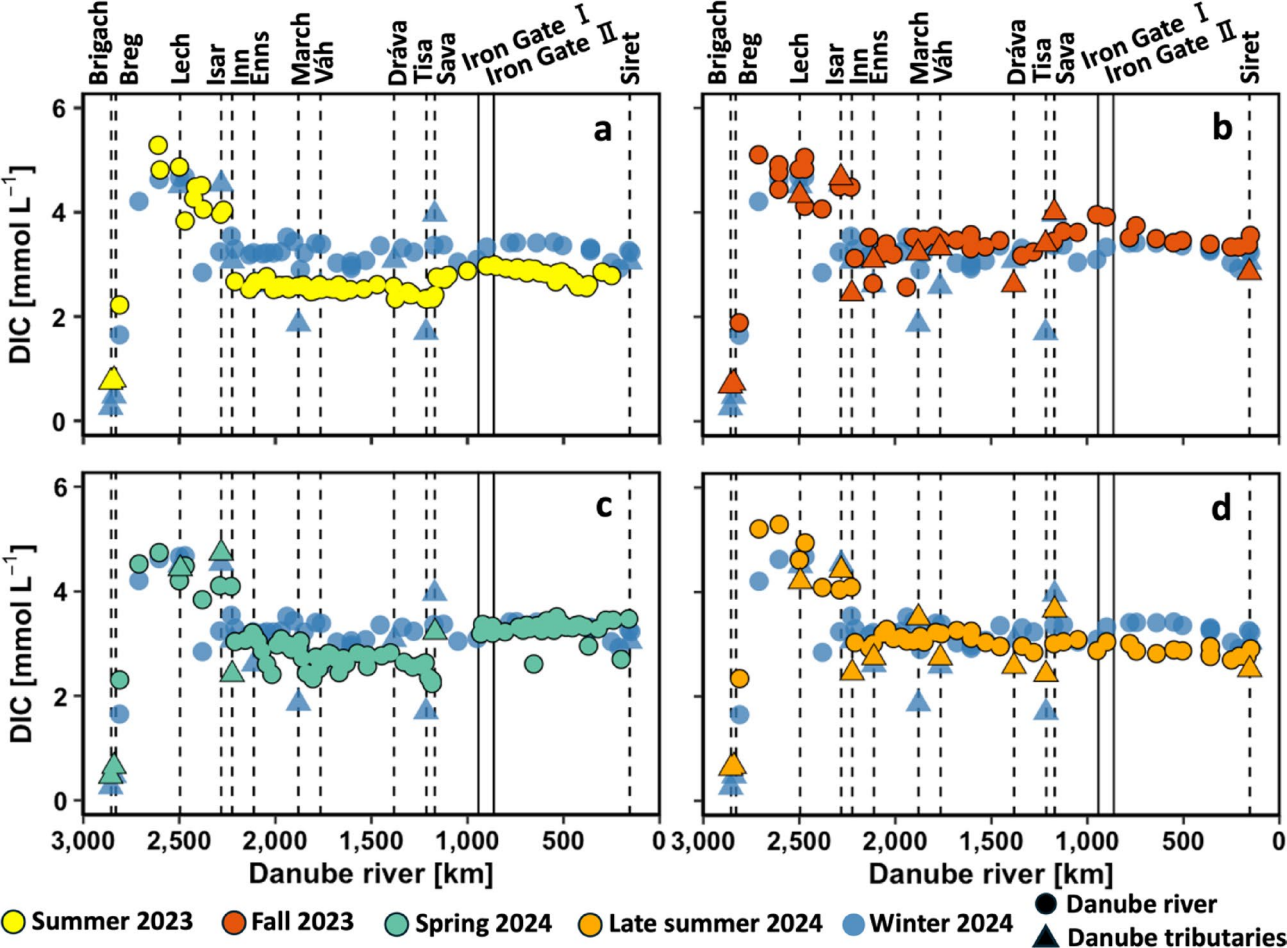
Dissolved inorganic carbon (DIC) concentrations (mmol L^-1^ ) along the Danube River. Distances are in river-kilometers from the Black Sea (0 km), with the Brigach and Breg headwaters located at 2,857 and 2,840 km, respectively. Each panel compares the winter conditions with those of the other seasons: (**a**) summer, (**b**) fall, (**c**) spring, and (**d**) late summer. Circles represent main river samples, and triangles represent tributary inputs. Standard errors are smaller than the symbol size. The dotted vertical lines mark confluences of major tributaries, and the solid vertical lines mark the Iron Gate I and II hydropower dams.

**Fig. 3:**
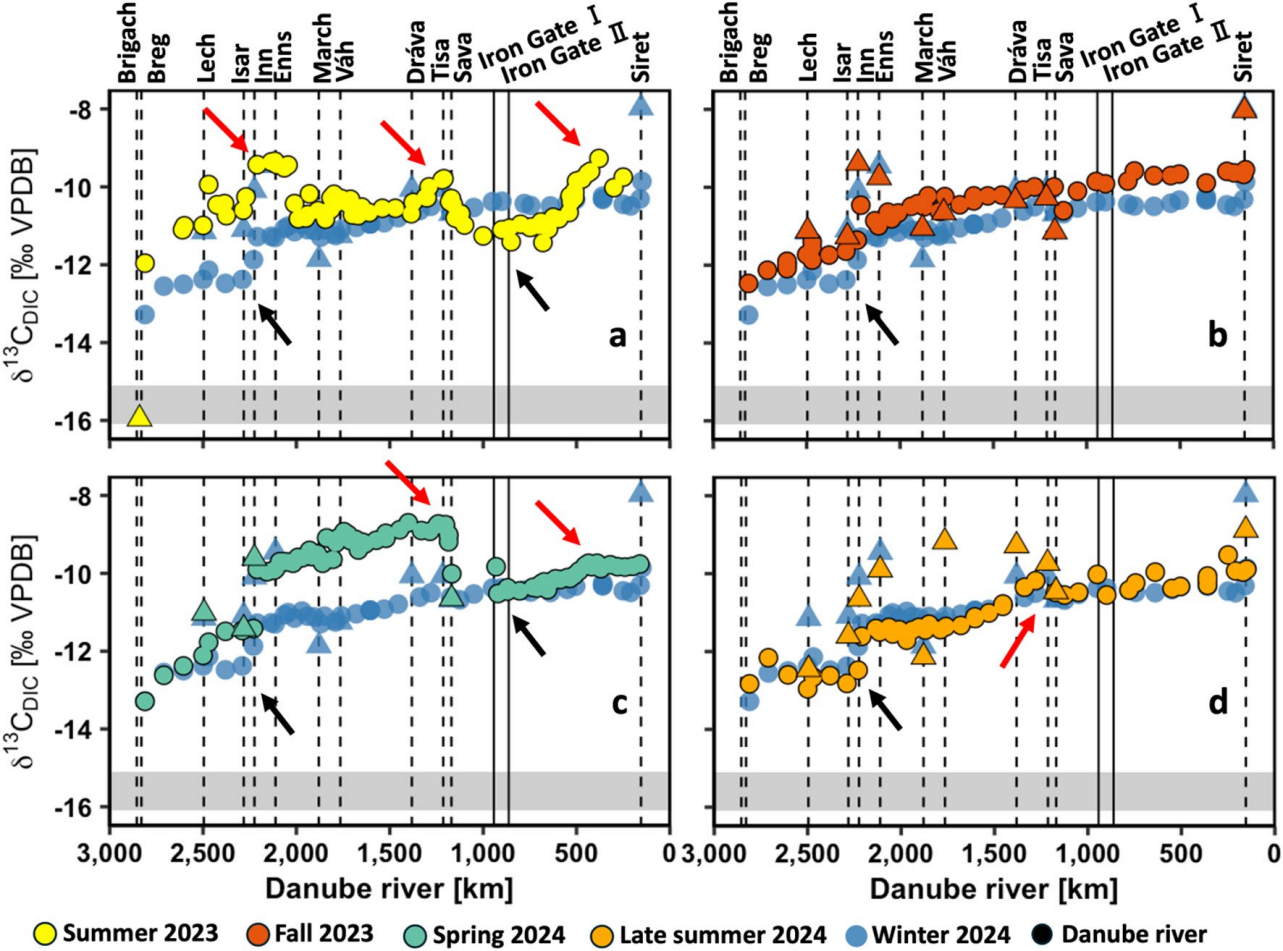
Stable isotopes of dissolved inorganic carbon (*δ*^13^C_DIC_) along the Danube River. Distances are in river-kilometers from the Black Sea (0 km), with the Brigach and Breg headwaters located at 2,857 and 2,840 km, respectively. Each panel compares winter conditions with one additional season: (**a**) summer, (**b**) fall, (**c**) spring, and (**d**) late summer. Circles represent main stem samples, and triangles represent tributaries. Standard error bars are smaller than symbol sizes. Dotted vertical lines mark confluences of major tributaries, whereas solid vertical lines mark the Iron Gate I and II hydropower dams. The gray horizontal bars show the range for expected *δ*^13^C_DIC_ values in groundwater. The red arrows mark the *δ*^13^C_DIC_ maxima and the black arrows mark the *δ*^13^C_DIC_ minima.

**Fig. 4:**
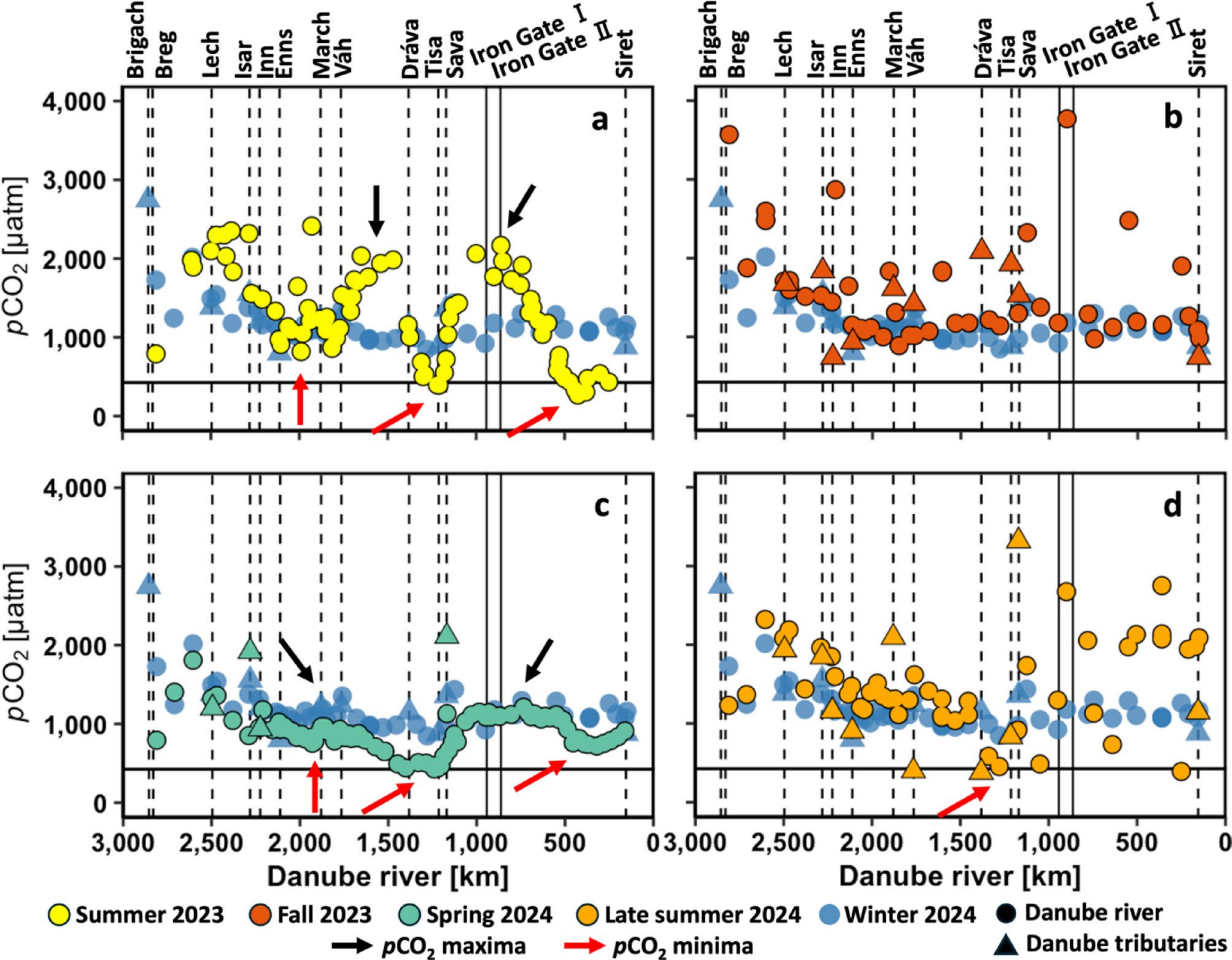
*p*CO_2__(aq)_ values [µatm] along the Danube River. Distances are in river-kilometers from the Black Sea (0 km), with the Brigach and Breg headwaters located at 2,857 and 2,840 km, respectively. Each panel compares winter conditions with one selected season: (**a**) summer, (**b**) fall, (**c**) spring, and (**d**) late summer. Circles represent main river samples, and triangles represent tributary inputs. Standard error bars are smaller than the symbol size. Dotted vertical lines mark confluences of major tributaries, whereas the solid vertical lines mark the Iron Gate I and II hydropower dams. The horizontal solid black line at 421 ± 1 µatm represents the current average value of atmospheric *p*CO_2_. Red arrows mark the *p*CO_2_ minima, and the black arrows mark the *p*CO_2_ maxima.

To investigate the seasonal dynamics of the Danube carbon cycle, DIC concentrations and their *δ*^13^C_DIC_ values, as well as calculated *p*CO_2(aq)_ concentrations were analyzed along the Danube River for each of the five sampling campaigns described above. Because winter conditions in the Danube were characterized by low temperatures and reduced light availability^20^, factors that generally constrain in-stream photosynthesis in river ecosystems, we used the winter sampling campaign as a reference for direct comparison (Figs. 2, 3 and 4).

Along the main stem of the Danube, DIC concentrations exhibited a consistent longitudinal pattern across all seasons, with values ranging from 0.3 to 5.3 mmol L^− 1^ (Fig. 2a–d). The lowest concentrations were observed in the Brigach and Breg headwater streams. These values rapidly increased shortly after their confluence where the highest values were recorded. From this point onwards at km ∼2,610 km, DIC concentrations gradually decreased downstream and remained largely stable after the inflow of the Inn River (∼2,225 km). In addition to these general spatial trends, minor seasonal differences became evident. The spring, late summer, fall and winter samples presented broadly similar DIC spatial patterns (Fig. 2b–d), whereas samples from summer presented the strongest deviation from the otherwise consistent annual pattern (Fig. 2a).

Summer and late summer presented elevated DIC levels, which exceeded those of the winter in the upper Danube (Fig. 2a, d). In contrast, downstream of the Inn, the summer DIC concentrations were 0.5 to 1 mmol L^− 1^ lower than those of winter (∼3.2 mmol L^− 1^), whereas the late summer values approached those of winter.

DIC stable isotope values (*δ*^13^C_DIC_) ranged from  -13.3 to -8.7‰ across all seasons (Fig. 3), consistent with data found by Pawellek and Veizer^17^ . The two headwater streams (Brigach and Breg) were excluded from this range and from Fig. 3 because of their more negative compositions reached  -23.2‰ and  -18.6‰, respectively.

In winter, the *δ*^13^C_DIC_ values revealed spatially coherent downstream enrichment along the main stem of the Danube, with a distinct ∼1‰ increase at the Inn confluence (black arrow in Fig. 3a–d). This input was superimposed on the overall positive downstream trend.

In contrast, summer displayed stronger longitudinal variabilities with several local extrema (Fig. 3a). Three pronounced regional maxima occurred in the upper, middle, and lower Danube (red arrows), along with local minima (black arrows).

The fall transect exhibited a smooth and systematic downstream increase. However, its values were on average 0.5‰ more positive than those of winter (Fig. 3b).

During spring, *δ*^13^C_DIC_ increased again downstream, but two major shifts were obvious (Fig. 3c): (i) a ∼1.5‰ increase after the confluence of the Inn relative to winter, and (ii) a comparable decrease downstream of the Sava confluence (black arrows). Additionally, two subtle increases were obvious along the middle and lower Danube (red arrows; ∼1,220 km; ∼380 km, respectively).

In late summer, *δ*^13^C_DIC_ showed a continuous downstream increase that broadly aligned the winter pattern, with a similar increase as in winter at the Inn confluence (black arrow) and a modest middle Danube maximum (red arrow in Fig. 3d).

The determined aqueous partial pressures of *p*CO_2_ (*p*CO_2(aq)_) were compared with those from the atmosphere (*p*CO_2(air)_) to identify oversaturated (*p*CO_2(aq)_ > *p*CO_2(air)_) and undersaturated (*p*CO_2(aq)_ < *p*CO_2(air)_) river sections. We used this comparison also as differences in the calculation of CO_2_ fluxes (*F*_CO2_) from the river (Supplementary Information I). The mean atmospheric *p*CO_2(air)_ during all sampling campaigns was assumed with 421 ± 1 µatm, on the basis of the globally averaged monthly mean data ( https://gml.noaa.gov/webdata/ccgg/trends/co2/co2_mm_gl.txtlfor September 2025.

Along the Danube, the *p*CO_2(aq)_ values exhibited pronounced spatial and seasonal variabilities and ranged from below atmospheric equilibrium (∼266 µatm) to a maximum of ∼3,770 µatm. (Fig. 4). Overall, the river remained predominantly oversaturated, and reflected continuous outgassing along its course throughout all seasons. Relationships between log_10_*p*CO_2_ and *δ*^13^C_DIC_ were also evaluated (Supplementary Information II Fig. S4) showing a weak tendency for *δ*^13^C_DIC_ to increase as *p*CO_2_ decreased. This trend was most visible during spring and summer, while strong scatter limited its interpretability for the entire basin.

In winter, the *p*CO_2(aq)_ progressively decreased downstream and stabilized at ∼1,000 µatm (at ∼2,060 km) after the confluence with the Enns River, with subsequent minor variations along the remaining river course (Fig. 4a–d).

During summer, *p*CO_2(aq)_ exhibited a pronounced longitudinal variability along the Danube River, which was characterized by three distinct minima (red arrows). In addition, two short undersaturated sections were observed in the middle (∼1,210 km; ∼393 µatm) and lower Danube (∼425 km; ∼266 µatm; Fig. 4a). These minima were interrupted by sharp increases in *p*CO_2(aq)_ up to ∼2,000 µatm (black arrows; ∼1,650 km and ∼860 km, respectively).

The spatial pattern of the fall season was broadly similar to that of the winter season, with occasional short-term increases or decreases in *p*CO_2(aq)_ and slightly elevated concentrations toward the lower reaches (Fig. 4b).

During spring, *p*CO_2(aq)_ followed a longitudinal pattern, similar to that in summer, but the variability was less pronounced (Fig. 4c). Overall spring samples showed the same sequence of minima (red arrows) and maxima (black arrows) as during summer. The *p*CO_2(aq)_ initially decreased downstream and reached a minimum of ∼750 µatm (∼1,930 km), followed by two further minima in the middle (∼1,240 km; ∼426 µatm) and the lower Danube (∼320 km; ∼718 µatm). These minima were separated by modest increases in *p*CO_2(aq)_ to ∼1,000 µatm that were comparable to winter levels.

In late summer, *p*CO_2(aq)_ exhibited a spatial pattern that was again comparable to that of winter, with isolated elevated values along the main stem (Fig. 4d). A weak minimum was still discernible in the middle Danube (∼1,280 km; ∼452 µatm), where concentrations approached near-equilibrium values.

Calculated *F*_CO2_ fluxes followed the same seasonal pattern as *p*CO_2(aq)_. Maximum fluxes occurred during fall (1.993 Gg C d⁻¹) and late summer (1.950 Gg C d⁻¹), consistent with elevated *p*CO_2__(aq)_ along the river. Summer fluxes were intermediate (1.513 Gg C d⁻¹), while winter (1.389 Gg C d⁻¹) and spring (0.975 Gg C d⁻¹) fluxes were lower, corresponding to periods of more uniform or locally reduced *p*CO_2__(aq)_ (Supplementary Information I Table 1).

## Discussion

Spatial variations in DIC concentrations along the Danube River reflect the interplay between bedrock weathering, subsequent groundwater input, and hydrological mixing along the river continuum. Upstream of the Inn confluence (∼2,225 km from the mouth), DIC systematically increased across all seasons (Fig. 2a–d) and marked the transition from silicate-dominated headwaters of the Black Forest to the carbonate-rich upper Danube region^49^ . A particularly steep increase to ∼5.3 mmol L^− 1^ occurred within the karstic section near Immendingen and Fridingen (river km ∼2,740 to ∼2,720), where enhanced interaction with carbonate aquifers led to elevated concentrations of weathering-derived DIC^30, 50^. This karst-mediated groundwater recharge likely accounted for much of the pronounced DIC increase between the headwaters and river km ∼2,600 and coincided with the dominant role of carbonate bedrock in supplying geological DIC to ground and surface waters in karst terrains^51, 52, 53^ . Parallel increases in HCO_3_^−^ and Ca^2+^ concentrations further confirm the strong carbonate-weathering control on DIC dynamics in the upper river (Supplementary Information II Figs. S1, S2): This trend also coincides with characteristic geochemical signals of carbonate dissolution observed in other river systems worldwide^41, 54^.

Downstream of this karst-dominated section, DIC concentrations gradually declined toward the confluence of the Inn River, primarily after confluences of tributaries Lech and Isar, both of which transport substantially lower DIC waters (4.4 ± 0.1 mmol L-^1^ and 4.6 ± 0.1 mmol L-^1^, respectively; Fig. 1; ^31, 32, 33^). The confluence of the Inn caused further decreases of DIC concentrations. Although the Inn River mostly drains carbonate terrains, its catchment is also influenced by glacial meltwater and rapid runoff from the Alps. These characteristics limit carbonate dissolution and result in comparatively low DIC concentrations (2.6 ± 0.4 mmol L^− 1^ DIC). Given that the discharge of the Inn is comparable to that of the Danube, this inflow exerts a pronounced dilution effect across all seasons (Supplementary Information II Fig. S3).

Downstream of the Inn, the DIC variability sharply decreased, and concentrations remained nearly uniform towards the river mouth. This homogenization reflects large-scale hydrological integration and the progressive admixture by groundwater and tributary inputs from an increasingly complex geological basin (Supplementary Information II Fig. S3; ^30, 36^). With increasing catchment size, lithologically diverse subbasins contribute to an average DIC signal that is stabilized by a large, groundwater-sustained pool with weathering of carbonates and silicates. This homogenized input effectively buffers DIC concentrations against local geochemical variabilities. Superimposed on this basin-scale DIC trend, minor deviations occur at major tributary confluences. For example, the Tisa River introduces low DIC water that transposes into a slight decrease in DIC in the middle of the Danube. In contrast, the Sava River drains carbonate-rich terrains and contributes higher-DIC waters. This input in turn leads to a measurable increase in DIC downstream of the Tisa confluence (Figs. 1 and 2; ^30^).

Moderate seasonal variations were superimposed on this longitudinal pattern. For example, DIC concentrations were lowest in summer and late summer (Fig. 2). These decreases could have been caused by DIC removal via photosynthesis. In contrast, fall and winter exhibited nearly identical DIC levels despite pronounced differences in discharge (Supplementary Information II Fig. S3). This similarity likely indicates a stable, basin-wide mixed groundwater and tributary signal with weathering-derived DIC along the river continuum.

Overall, the longitudinal DIC profile primarily reflects the control of a mixture of carbonate and silicate weathering together with progressive hydrological mixing. The downstream stabilization of DIC highlights the increasing dominance of hydrogeological integration and buffering by groundwater with its weathering products. This process dampens the expression of local and regional geochemical DIC variabilities in this large river system.

In contrast to the uniform DIC concentrations, *δ*^13^C_DIC_ provided a more sensitive tracer of carbon sources, sinks and turnover along the Danube (Fig. 3a–d). Across all seasons, the *δ*^13^C_DIC_ values increased from source to mouth. This trend coincides with progressive loss of ^12^C-enriched CO_2_ during degassing, but similar *δSUPERSCRIPT 13 *13C_DIC_ values may also arise from carbonate weathering under open-system conditions involving soil CO_2_. To interpret this longitudinal enrichment, *δ*^13^C_DIC_ values must be evaluated relative to the isotope value of soil-derived CO_2_ and its progression into groundwater. In temperate catchments dominated by C_3_ vegetation, soil-respired CO_2_ assumes the value of the original biomass 55. In the Danube catchment we found *δ*^13^C_DOC_ values between  -28 and  -29‰ that reflect the organic matter also in soils. During diffusive transport within the soil pore space, the freshly produced CO_2_ becomes enriched in ^13^C by approx. +4.4‰ due to kinetic fractionation. This process yields residual soil CO_2_ values between  -24.6 and  -23.6‰ ^44^. Subsequent equilibration of this diffusively enriched soil CO_2_ causes an additional temperature- and pH-dependent equilibrium fractionation of about + 8.5‰ between gaseous CO_2_ and DIC at typical groundwater temperatures of 12 °C in the Danube Basin. Here individual fractionations between CO_2(g)_ and each DIC species (CO₂_(aq)_, HCO₃⁻, and CO₃²⁻) have to be considered.

Under these conditions, *δ*13C_DIC_ values between  -15.1 and  -16.1‰ are expected for this equilibrium (gray bar in Fig.3: ^43, 56^). These values were calculated as the weighted average of the pH- and T-dependent distribution of DIC species (CO_2(aq)*_, HCO_3_^−^ and CO_3_^2−^), each associated with its respective equilibrium isotope fractionations (ε), following Myrttinen et al. ^45^. Accordingly, *δ*¹³C_DIC_ represents a bulk signal that integrates species-specific fractionation and their relative abundances under given physicochemical conditions. The absence of such typical groundwater values in the Danube indicates rapid overprinting by CO_2_ degassing, once groundwater enters the river and dissolved CO_2_ becomes exposed to open-channel gas exchange. In addition to degassing, photosynthetic uptake of ^12^CO_2_ can further enrich *δ*^13^C_DIC_ in ^13^C ^57^.

The above interpretation assumes that groundwater derived DIC can be approximated by isotope equilibration between soil CO₂ and DIC. This conceptual framework is expected to be the most applicable where sufficient contact times permit extensive equilibration along groundwater flow paths. However, the degree of equilibration likely varies across the heterogeneous hydrogeological settings of the Danube Basin. In particular, rapid infiltration and flow though karst systems may limit equilibration and preserve a stronger impact of weathering derived carbon sources. Furthermore, the relative contributions of groundwater sources with different residence times, flow paths, and lithological settings are expected to vary under different hydrological conditions. Consequently, the equilibrium range presented here should be regarded as a first-order reference endmember rather than a universally applicable groundwater signature. In the absence of basin-scale groundwater isotope data, this approximation nevertheless provides a useful framework for evaluating the downstream evolution of riverine *δ*^13^C_DIC_.

The spring *δ*^13^C_DIC_ values revealed a broadly similar downstream enrichment pattern to that of winter but featured two pronounced anomalies (Fig. 3c). At the Inn confluence (black arrow), the *δ*^13^C_DIC_ increased sharply by ∼1.5‰ to a value of -9.9‰. This increase reflects the input of low-alkalinity, low-DIC, and alpine glacially derived waters that must have been subjected to CO_2_ outgassing during steep, turbulent flow (Supplementary Information II Fig. S1). During spring melt, high discharge of the Inn River often exceeds that of the Danube main stem (Supplementary Information II Fig. S3c) and allows the Inn´s isotope signal to dominate downstream of the confluence. Comparable patterns have been reported for other large rivers; for instance, Striegl et al. ^58^ observed that DIC concentration and *δ*^13^C_DIC_ in the Yukon River were strongly affected by dissolution of suspended carbonates in glacial meltwaters. Further downstream, the Sava introduces DIC-concentrated water that is more enriched in ^12^C (Supplementary Information II Fig. S1; Fig. 2). This input must have caused *δ*^13^C_DIC_ values that are closer to those of winter (black arrow in Fig. 3c). Despite draining limestone-rich uplands, the Sava´s chemically buffered, low-turbulence water underwent little CO_2_ degassing and thus likely retained low *δ*^13^C_DIC_ values^59, 60^ . The fact that the Sava contributes up to ∼25% of total Danube discharge^30^ , its inflow rapidly dilutes and suppresses the upstream signal. Moreover, influences of photosynthesis likely remained modest during spring because of the relatively low temperatures and limited availability of light. Nonetheless, minor increases in the middle and lower reaches of the river (red arrows) indicate early onsets of biological activities by photosynthesis^61^ .

During summer, *δ*^13^C_DIC_ exhibited the strongest deviations from those of winter, with pronounced maxima in the upper, middle, and lower Danube (Fig. 3a). In the upper reach, *δ*^13^C_DIC_ increased again at the Inn confluence, and reached a value of -9.4‰. This enrichment reflects a combination of intensified photosynthetic ^12^CO_2_ uptake under warm, light-intensive conditions and the continued input of ^13^C-enriched meltwater from the Inn River (black and red arrows in Fig. 3a). Despite the persistence of alpine contributions, the relative discharge of the Inn is considerably lower than that of the Danube compared to spring and likely reduced its hydrological dominance and preserved a detectable isotope imprint. The reduced magnitude of the summer *δ*^13^C_DIC_ shift therefore likely reflects smaller tributary influence rather than a less enriched source^62^ . Downstream of the Inn-Enns section, *δ*^13^C_DIC_ gradually declined toward winter baseline values as groundwater-derived DIC became more prominent under generally low summer discharge. Further downstream, renewed *δ*^13^C_DIC_ increases in the middle and lower Danube indicate localized in-stream photosynthetic enrichment (red arrows), as also observed in other studies of large rivers where metabolism and land use influence carbon isotope composition^63^ . These sections coincide with reaches that were previously identified as areas of elevated primary productivity (e.g., ^20, 64, 65^). These biologically driven isotope maxima were sharply interrupted downstream of the Sava confluence, where the inflow of high DIC, and low turbulence water induced isotope dilution, driving *δ*^13^C_DIC_ values below the winter baseline (black arrow). This effect was particularly pronounced in summer, when reduced Danube discharge allowed the carbon dynamics of major tributaries to exert a stronger influence. Comparable processes have been observed in other large rivers, where tributary inflows interact with carbonate-rich sediments, leading to dissolution and modification along the river continuum^66^ . Similar influences by groundwater are also possible but cannot be separated from tributary inputs with the current dataset.

During late summer and fall, *δ*^13^C_DIC_ broadly followed the winter baseline as biological enrichment weakened with decreasing temperature and light availability. Reduced discharge and longer water residence times likely promoted continued but slow CO_2_ outgassing, thus leading to slight but persistent ^13^C enrichment in the remaining DIC pool during both seasons. These hydrologically stable, low turbulence conditions favor carbonate system buffering and gas exchange over biological turnover and mark the seasonal transition from biologically driven isotope variability towards a predominantly groundwater- carbonate controlled carbon regime of the river with subsequent degassing.

While these processes consistently explain the overall downstream enrichment of *δ*^13^C_DIC_, the relative contribution of different carbon sources remains less well constrained. A potential additional contribution may arise from C_4_ vegetation (-12.5‰) ^43^, which has regionally expanded in parts of eastern Europe and may increase C_4_-derived carbon inputs to soils and groundwater towards the lower Danube^67^.

**Fig. 5:**
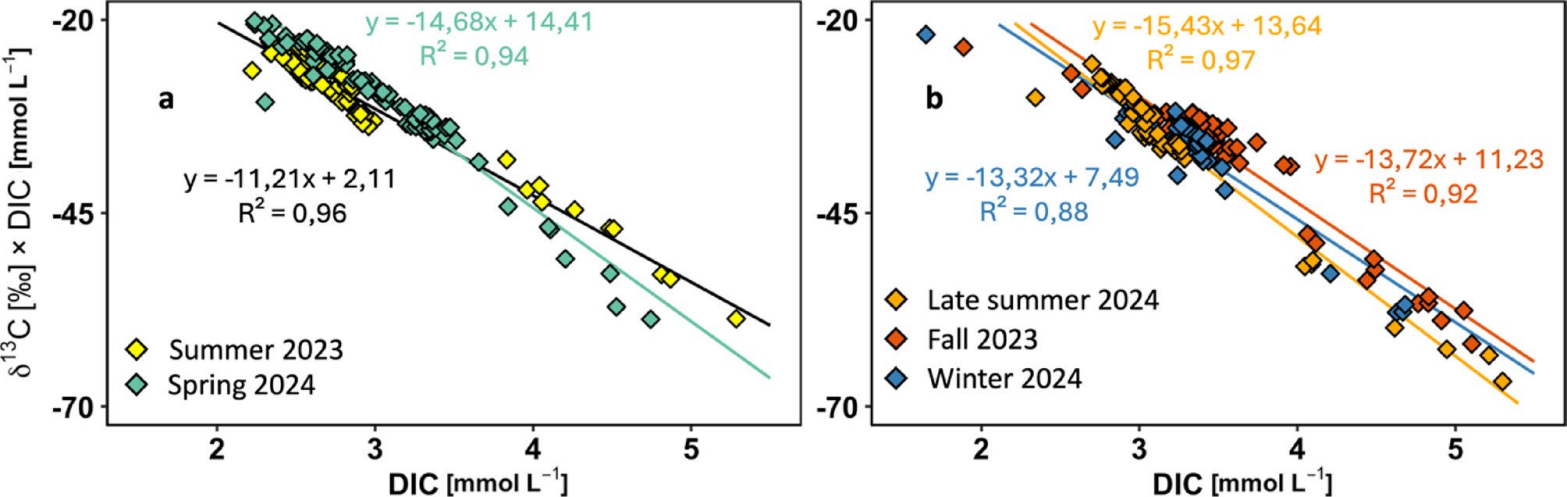
Miller-Tans plots of DIC versus *δ*¹³C_DIC_ for each season (**a**) spring and summer, and (**b**) late summer, fall, and winter. The slope of each dataset represents the *δ*¹³C_DIC_ signature of groundwater inputs.

To evaluate this effect, we applied a Miller-Tans plot (Fig. 5), where the slopes of seasonal regressions the input *δ*^13^C_DIC_ signal and corresponds to the *δ*^13^C_DIC_ as it originally entered the river from its groundwater source^48, 53^ . The derived *δ*^13^C_DIC_ source values (-11.2 to -15.4‰) deviate from the expected equilibrium range for purely C_3_-derived soil CO₂ in the Danube (-15.1 to -16.1‰), particularly during summer, indicating an additional heavier carbon source. Using a two-endmember mixing model based on C_3_- and C_4_-derived DIC inputs (Eq. 4), we estimate that C_4_ contributions may reach up to ~ 27% during summer, while remaining substantially lower during fall, winter, spring and late summer (Table 1). The applied endmembers assume idealized equilibrium conditions and do not explicitly account for spatial variability in land use, groundwater flow paths, or overlapping in-stream processes such as CO₂ degassing and photosynthesis.

**Table 1:** Estimated relative contributions of groundwater influenced by C_3_ and C_4_ vegetation, calculated using the two-endmember mixing model (Eq. 4).

Season	C_3_ [%]	C_4_ [%]
Summer 2023	72.8	27.2
Fall 2023	88.5	11.5
Winter 2024	86.0	14.0
Spring 2024	94.5	5.5
Late summer 2024	99.2	0.8

Seasonal patterns indicate the highest C_4_ contributions during summer, with substantially lower values during the remaining seasons. Minor inconsistencies between campaigns likely reflect uncertainties in the mixing approach as well as temporal variability in groundwater flow paths and source contributions.

Despite this detectable influence, C_3_-derived carbon clearly dominates at the basin scale. This is supported both by consistently low *δ*¹³C_DOC_ values (-28 to -29‰), reflecting the prevailing organic matter composition of the catchment, and by seasonal C_3_ contributions, which consistently exceed 70% (Table 1).

The above considerations rely on a simplified two-endmember mixing model that distinguishes between DIC ultimately derived from C_3_ or C_4_-dominated vegetation. Consequently, the estimated contributions should be interpreted as indicative and as an upper-bound approximation rather than strictly quantitative source apportionments. A more comprehensive source framework could additionally distinguish between carbonate- and silicate-weathering contributions associated with both vegetation types. Such an approach would require independent constraints on groundwater endmembers, lithology-specific isotope signatures, and temporally variable groundwater flow paths. However, these parameters are not available for the present basin-scale dataset. Therefore, the two-endmember model should be viewed as a first-order approximation that provides insight into the potential influence of C_4_-derived carbon on the Danube DIC budget rather than a unique source apportionment. Future groundwater-focused studies would be required to quantify the relative contributions of more complex source combinations and their variability under changing hydrological conditions.

Overall, *δ*^13^C_DIC_ integrates the cumulative effects of groundwater-derived carbon inputs that are rapidly overprinted by CO_2_ degassing. Additional influences include photosynthetic ^12^CO_2_ uptake and the admixture of major tributaries along the Danube continuum. The progressively more positive *δ*^13^C_DIC_ primarily reflect efficient CO_2_ degassing, whereas sustained groundwater inputs and carbonate system buffering offer a basin-scale counterbalance to these processes. Superimposed spatial anomalies at major confluences and influences by C_4_ plants highlight the hydrochemical heterogeneity of this large fluvial system. Other studies have observed similar enrichments in *δ*^13^C_DIC,_ though at smaller scales and in headwater regions, emphasizing that such patterns are common feature of large river systems^68, 69, 70^ .

The consistently elevated *p*CO_2(aq)_ values along the Danube River confirm that CO_2_ degassing is a basin-wide and persistent process (Fig. 4a–d). Across all seasons, most *p*CO_2(aq)_ values exceeded atmospheric equilibrium value of 421 µatm. However, degassing intensities varied seasonally and during fall and winter, *p*CO_2(aq)_ values remained uniformly high along the river continuum and reflected efficient CO_2_ degassing under colder, enhanced turbulence, and limited biological conditions. In contrast, during spring, summer and late summer, several river sections even fell below atmospheric equilibrium (red arrows) and indicated localized and transient CO_2_ drawdown. These *p*CO_2(aq)_ anomalies spatially coincided with *δ*^13^C_DIC_ maxima in the middle and lower Danube and occurred in reaches characterized by reduced flow velocities and a lower river gradient. This river regulation by damming enhanced the sedimentation of suspended matter and led to reduced turbidity and increased light penetration. Together with sustained nutrient inputs from agricultural land use, these conditions likely favored phytoplankton development and elevated photosynthetic activity, which locally reduce *p*CO_2(aq)_. Comparable summer CO_2_ undersaturation has been reported for other large rivers, including the upper Mississippi River, where damming and nutrient inputs promote phytoplankton production^15^ , and the St. Lawrence River, where lake regulation and strong seasonal photosynthesis lead to undersaturated reaches during summer and fall^14^ .

Independent evidence for enhanced biological activity in these middle and lower Danube reaches with low *p*CO_2(aq)_ has been previously reported based on dissolved oxygen (DO) and its ^18^O/^16^O ratios^20^. While oxygen data are not re-evaluated here, their documented spatial patterns provide contextual support for the interpretation that photosynthetic activity contributes to localized CO_2_ uptake during favorable hydrological and thermal conditions. Notably, the magnitude and spatial expression of *p*CO_2(aq)_ variability differ fundamentally from those of oxygen-based indicators because of the contrasting pool sizes and buffering mechanisms of the underlying dissolved constituents. DO responds rapidly to short-term changes in photosynthesis, respiration, and degassing, owing to its relatively small reactive pool. In contrast, the DIC pool is one to two orders of magnitude larger and is strongly buffered by carbonate equilibrium, weathering with subsequent groundwater inflow, and continuous CO_2_ exchange with the atmosphere.

Consequently, *p*CO_2(aq)_ variability integrates biological CO_2_ uptake with physicochemical control, such as temperature-dependent solubility and carbonate-system buffering. This buffering explains why pronounced seasonal *p*CO_2(aq)_ fluctuations of up to ∼2,000 µatm can occur even when total DIC concentrations remain stable. Calculated fluxes of CO_2_ closely follow the seasonal *p*CO_2(aq)_ patterns. Higher fluxes in fall and late summer correspond to elevated *p*CO_₂(aq)_ along the river, while lower fluxes in spring and winter reflect periods of reduced or more uniform *p*CO_2__₂(aq)_ (Supplementary Information I). These fluxes provide a basin-wide measure of CO₂ outgassing that integrates the combined effects of degassing and localized biological activity, highlighting the Danube’s role as a persistent CO₂ source to the atmosphere.

## Conclusions

Our study provides a new basin-scale, multi-seasonal assessment of DIC in the Danube as its most important carbon phase. Data were combined with associated *δ*^13^C_DIC_ isotopes and dissolved gases (*p*CO_2(aq)_) to evaluate how weathering, hydrological mixing, tributary inputs, biological activity, land use and CO_2_-degassing interact to shape DIC dynamics in this river system.

DIC levels are primarily controlled by lithology and hydrology, with strong carbonate-weathering inputs in the upper basin and progressive downstream homogenization driven by large-scale hydrogeological integration. In contrast, *δ*^13^C_DIC_ and *p*CO_2(aq)_ provide more sensitive indicators of additional carbon input by groundwater and subsequent turnover and loss processes. Input by different forms of land use with C_3_ and C_4_ cultivation could be evaluated via a mass balance and indicated C_4_ signals during summer. However, this evaluation relies on a simplified two component mass-balance and could be refined in more detailed small-scale studies that include detailed groundwater monitoring.

Although persistent CO_2_ supersaturation and atmospheric degassing characterize the Danube River, photosynthetic CO_2_ uptake can produce pronounced and spatially as well as seasonally limited deviations, particularly during summer. Calculated CO₂ fluxes (0.975–1.993 Gg C d^−^¹) followed these seasonal patterns, confirming that the Danube is a persistent CO₂ source, with slightly lower fluxes in spring and localized reductions during summer reflecting temporary biological uptake and increased in-stream algae productivity. These areas and periods of CO_2_ drawdown reflect increased in-stream algae productivity but remain superimposed on strongly buffered weathering systems dominated by groundwater-derived alkalinity. This shows that for the riverine carbon cycle biological effects are largely dampened, and homogeneous DIC concentrations and overall CO_2_ supersaturation remain dominant along most of the river continuum.

In summary, our results highlight the primary role of hydrogeological integration, land use effects and geochemical buffering in regulating carbon input, turnover and loss from the Danube as a typical representative for a complex and large river system. Nonetheless, we were also able to show secondary influences of biological processes under favorable seasonal conditions. The observed decoupling between nearly homogeneous DIC concentrations and more variable *δ*^13^C_DIC_ and *p*CO_2(aq)_ values demonstrates that land use and in-river secondary biological processes can locally and seasonally modify river carbon dynamics. These effects become best detectable when concentration data are combined with stable isotope constraints and CO_2_ system parameters.

From a hydrological perspective, these findings emphasize the importance to integrate weathering, land use, stable isotopes, and gas-exchange as indicators to interpret riverine carbon cycling under changing environmental conditions. Future work should focus on resolving groundwater end-member compositions, discharge variability, and continuous observation of *p*CO_2_ dynamics to better constrain how climate- and land-use-driven hydrological changes may influence carbon cycling and CO_2_ degassing in large river systems.

## Supplementary Information

Below is the link to the electronic supplementary material.

Supplementary Material 1

Supplementary Material 2

## Data Availability

The dataset is stored and available on PANGAEA under https://doi.pangaea.de/10.1594/PANGAEA.993653^71^.
