# Supplementary material for: Geological, land use and biological influences on carbon cycling and CO2 degassing in the Danube River
Source: Sci Rep. 2026 Jun 26;16:19624. doi: 10.1038/s41598-026-59715-0 (PMC13309551; doi:10.1038/s41598-026-59715-0)
Supplement: Supplementary file 1 — Supplementary Material 1 [file 41598_2026_59715_MOESM1_ESM.docx]

**SuppLementary Information I**

This file contains supporting online material that relates to the following publication:

Jan Maier^1^, Johannes A. C. Barth^1^

Geological, land use and biological influences on carbon cycling and CO_2_ degassing in the Danube River

**Author affiliations:**

^1^Department of Geography and Geosciences, Geozentrum Nordbayern, Friedrich-Alexander-Universität Erlangen-Nürnberg, Schlossgarten 5, 91054 Erlangen, Germany

*Correspondence to*: Jan Maier ([jan.m.maier@fau.de](mailto:jan.m.maier@fau.de))

# **1. Material and method**

# **1.1 CO_2_ flux calculations**

The air–water ﬂux of CO_2_ is expressed by Raymond et al **^1^**:

| $\boldsymbol{F}_{\boldsymbol{CO}\boldsymbol{2}}\boldsymbol{=k}* \left( {\mathbf{[}\boldsymbol{CO}}_{\boldsymbol{2}} \right]$_water_$- \left( {\mathbf{[}\boldsymbol{CO}}_{\boldsymbol{2}} \right]$_air_$)$ | (1) |
| --- | --- |

where *F*_CO₂_ represents the CO₂ flux (mmol m⁻² day⁻¹) across the air–water interface, ([CO_2_]) denotes the CO₂ concentration (mmol m⁻³) in water and air, and (k) is the gas transfer velocity (m day⁻¹). In this study, (k) was calculated as k600, i.e., the gas transfer velocity normalized to a Schmidt number of 600, which corresponds to CO₂ in freshwater at 20 °C according to Raymond et al **^1^**:

| $k_{600}= V* S* 2841+ 2.02$ | (2) |
| --- | --- |

where (V) is the stream velocity (m s⁻¹) and (S) is the dimensionless slope of the riverbed.

Table 1 provides an overview of the values used for calculating the gas transfer velocity (k_600_). Riverbed slopes were derived from Lászlóffy ^2^, while flow velocities and segment lengths were obtained from ICPDR ^3^, following the river segmentation proposed by Lászlóffy ^2^.

| **River section** | **Stream velocity [V] [**m s⁻¹**]** | **Slope [S] [-]** | **Distance from mouth [km]** |
| --- | --- | --- | --- |
| **Upper Danube** | 1.5 | 0.00063 | 2857 - 1838 |
| **Middle Danube** | 0.78 | 0.00006 | 1838 - 850 |
| **Lower Danube** | 0.81 | 0.00005 | 850 - 0 |

**Table 1: Parameters used for the calculation of gas transfer velocity (k_600_).**

For each river section (Upper Danube, Middle Danube, and Lower Danube), three width measurements were conducted: one at the sampling point and two at locations halfway to adjacent points. All measurements were taken perpendicular to the flow direction. River widths were determined using the measurement tool implemented in QGIS, based on a 1-meter resolution orthophoto provided by ESRI ^4^. The surface area of each river section was calculated by multiplying the mean river width by the corresponding section length, assuming a representative and spatially uniform width within each section.

Seasonal CO₂ fluxes (*F*_CO₂_) were calculated for representative sampling during each campaign.

| **Season** | ***F*_CO₂_ [Gg C d^-1^]** |
| --- | --- |
| Summer 2023 | 1.513 |
| Fall 2023 | 1.993 |
| Winter 2024 | 1.389 |
| Spring 2024 | 0.975 |
| Late summer 2024 | 1.950 |

**Table 2: Daily CO₂ fluxes (***F*_CO₂_**) for each sampling** **season.**

# **References**

1. Raymond, P. A. *et al.* Scaling the gas transfer velocity and hydraulic geometry in streams and small rivers. *Limnology and Oceanography: Fluids and Environments* **2**, 41–53 (2012).

2. Lászloffy W. Die Hydrographie der Donau. in *Liepolt R. (ed) Limnologie der Donau. Eine monographische Darstellung* 16–57 (I. Schweizerbart, Stuttgart, 1967).

3. Liška, I. *et al.* *Joint Danube Survey 4 Scientific Report: A Shared Analysis of the Danube River. Vienna: International Commission for the Protection of the Danube River-ICPDR*. (2021).

4. ESRI. World imagery map. Zugang via https://services.arcgisonline.com/ ArcGIS/rest/services/World_Imagery/MapServer (letzter Aufruf 03.07.2024). Preprint at (2021).
