# Supplementary material for: Geological, land use and biological influences on carbon cycling and CO2 degassing in the Danube River
Source: Sci Rep. 2026 Jun 26;16:19624. doi: 10.1038/s41598-026-59715-0 (PMC13309551; doi:10.1038/s41598-026-59715-0)
Supplement: Supplementary file 2 — Supplementary Material 2 [file 41598_2026_59715_MOESM2_ESM.docx]

**SuppLementary Information II**

This file contains supporting online material that relates to the following publication:

Jan Maier^1^, Johannes A. C. Barth^1^

Geological, land use and biological influences on carbon cycling and CO_2_ degassing in the Danube River

**Author affiliations:**

^1^Department of Geography and Geosciences, Geozentrum Nordbayern, Friedrich-Alexander-Universität Erlangen-Nürnberg, Schlossgarten 5, 91054 Erlangen, Germany

*Correspondence to*: Jan Maier ([jan.m.maier@fau.de](mailto:jan.m.maier@fau.de))


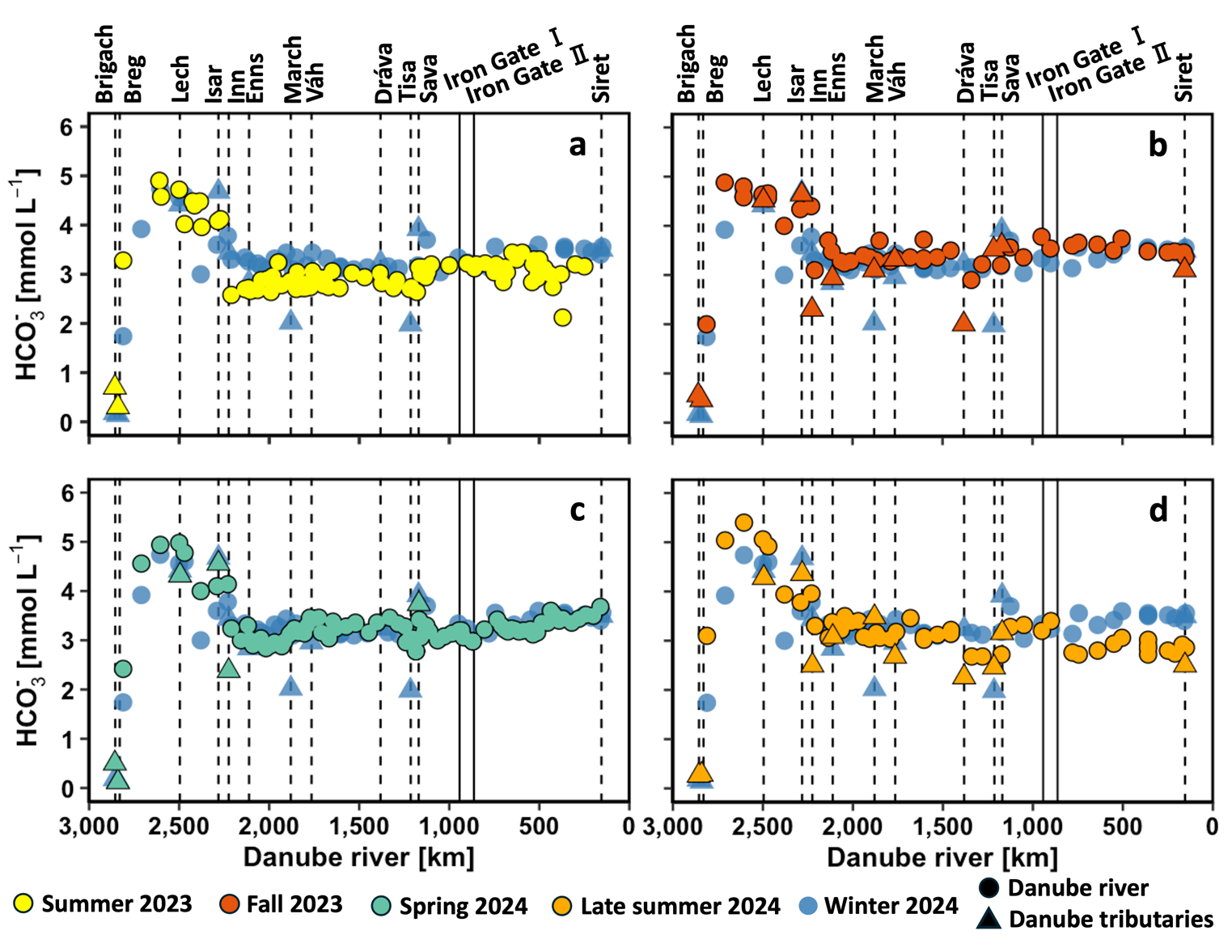
Figure S1. Bicarbonate (HCO_3_^-^) concentrations (mmol L^-1^) along the Danube River. Distances are in river-kilometers from the Black Sea (0 km), with the Brigach and Breg headwaters located at 2,857 and 2,840 km, respectively. Each panel compares winter conditions with the other seasons: (a) summer, (b) fall, (c) spring, and (d) late summer. Circles show main-stem samples and triangles indicate tributary inputs. Standard errors are smaller than the symbol size. Dotted vertical lines mark the confluence of major tributaries, while solid vertical lines mark the Iron Gate I and II hydropower dams.


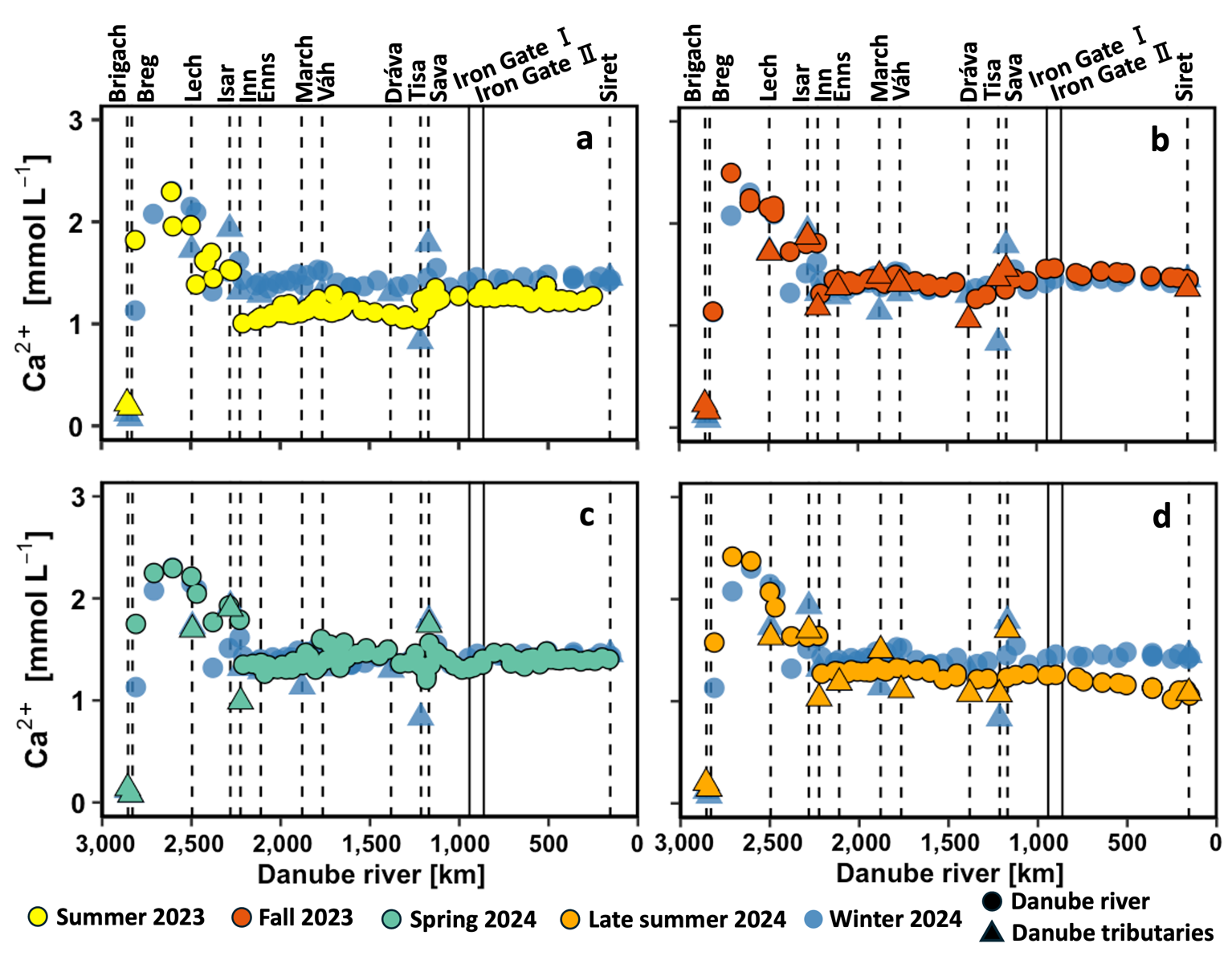


**Figure S2. Calcium (Ca^2+^) concentrations (mmol L^-1^) along the Danube River. Distances are in river-kilometers from the Black Sea (0 km), with the Brigach and Breg headwaters located at 2,857 and 2,840 km, respectively. Each panel compares winter conditions with the other seasons: (a) summer, (b) fall, (c) spring, and (d) late summer. Circles show main-stem samples and triangles indicate tributary inputs. Standard errors are smaller than the symbol size. Dotted vertical lines mark the confluence of major tributaries, while solid vertical lines mark the Iron Gate I and II hydropower dams.**

**
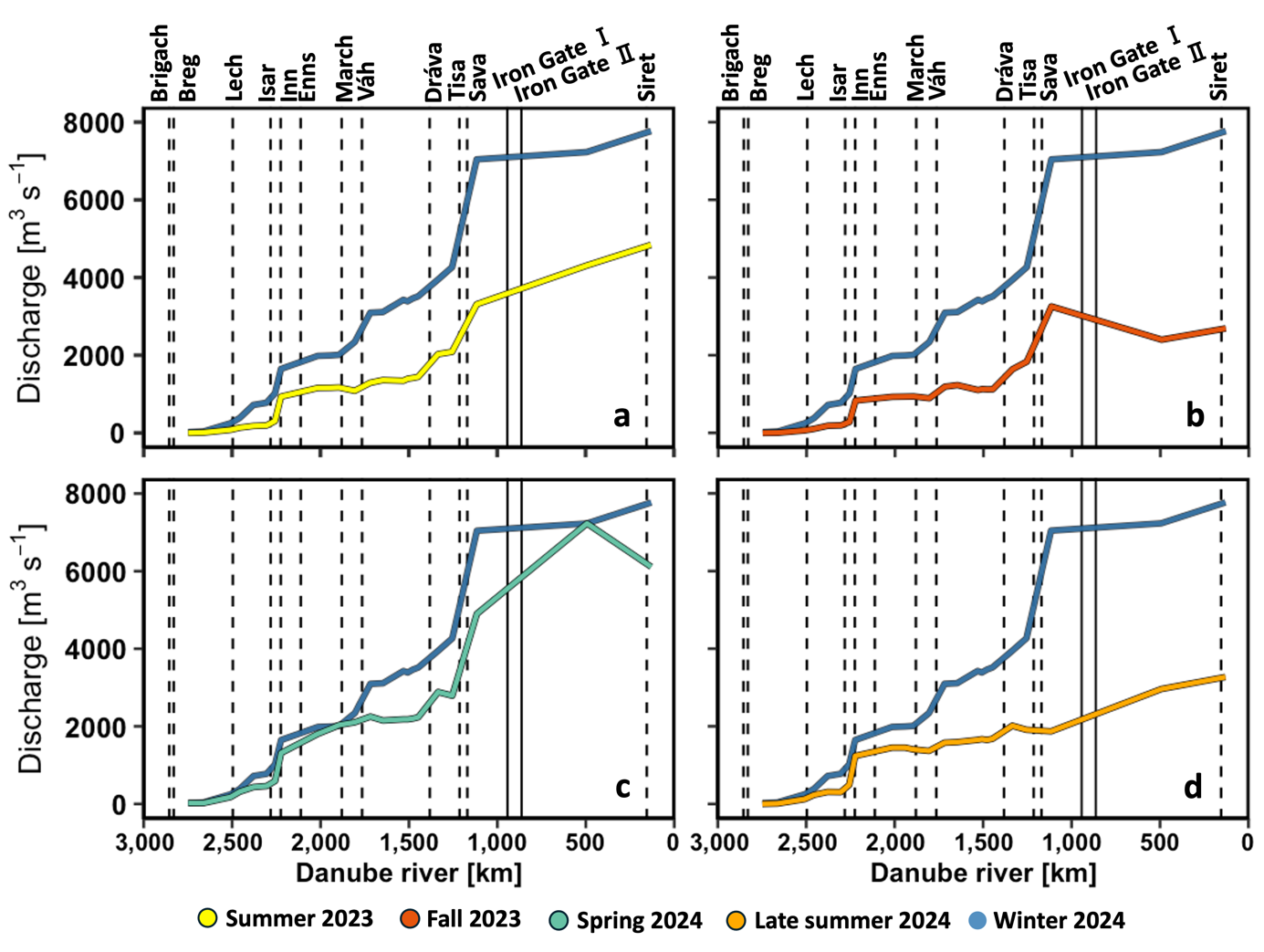
**

**Figure S3. Discharge data (m^3^ s^-1^) along the Danube River. Distances are in river-kilometers from the Black Sea (0 km), with the Brigach and Breg headwaters located at 2,857 and 2,840 km, respectively. Discharge data provided by <https://www.danubehis.org> (last access: 11 March 2025; ICPDR, 2024). Each panel compares winter conditions with the other seasons: (a) summer, (b) fall, (c) spring, and (d) late summer. Circles show main-stem samples and triangles indicate tributary inputs. Standard errors are smaller than the symbol size. Dotted vertical lines mark the confluence of major tributaries, while solid vertical lines mark the Iron Gate I and II hydropower dams.**


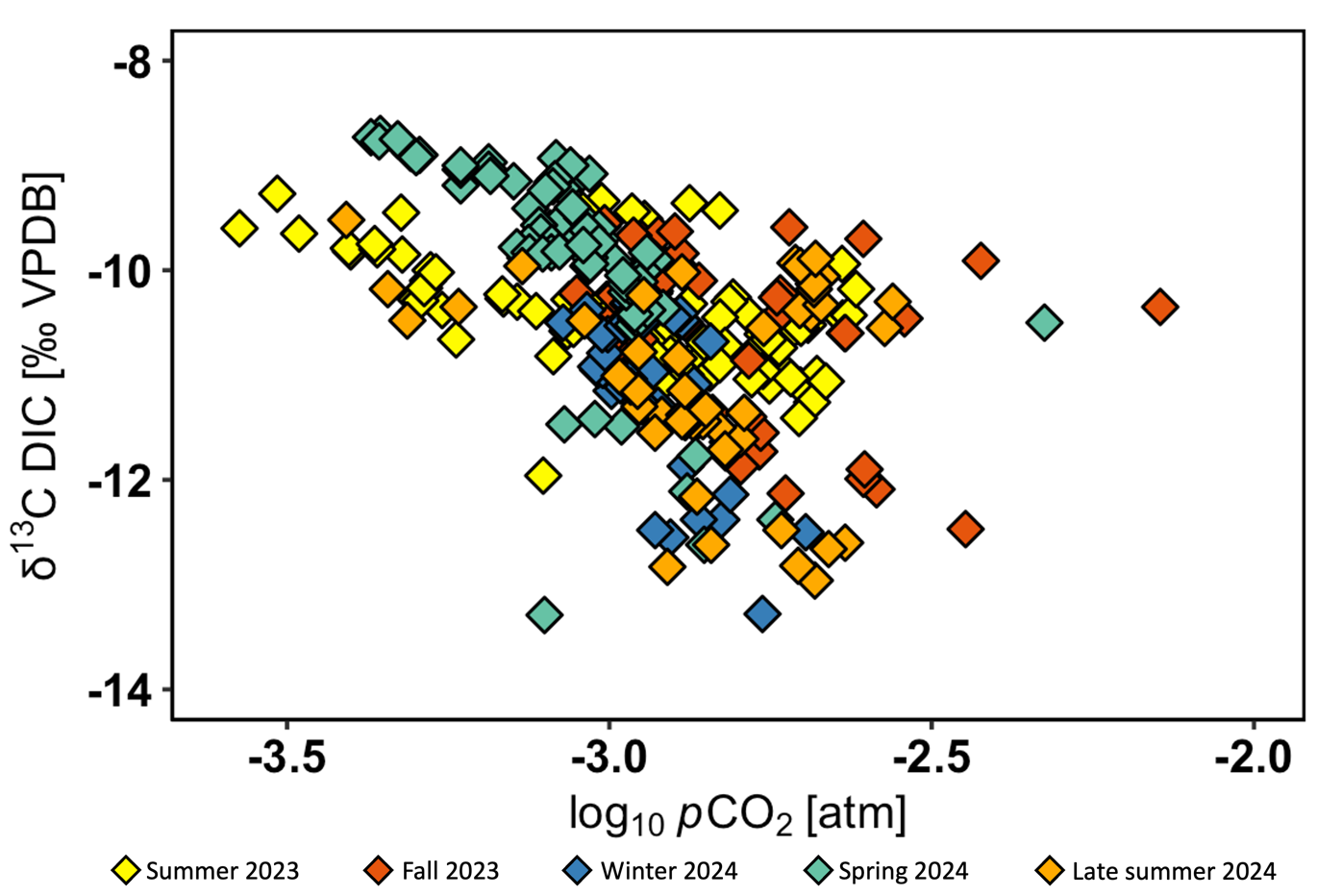


**Figure S4 Correlation plot for all Danube sampling locations of *δ*^13^C_DIC_ and log_10_ *p*CO_2_ [atm] in spring, summer, late summer, fall and winter.**
